# Authorization Patterns, Safety, and Effectiveness of Medical Cannabis in Quebec

**DOI:** 10.1089/can.2020.0140

**Published:** 2021-12-09

**Authors:** Maja Kalaba, Laura MacNair, Erica N. Peters, Graham M.L. Eglit, Lucile Rapin, Cynthia El Hage, Erin Prosk, Mark A. Ware

**Affiliations:** ^1^ Canopy Growth Corporation, Smiths Falls, Canada.; ^2^ Santé Cannabis, Westmount, Canada.

**Keywords:** medical cannabis, clinical practice, real-world data, symptom improvement, safety

## Abstract

**Introduction:** Despite increasing demand for data, little is known about the authorization patterns, safety, and effectiveness of medical cannabis products.

**Materials and Methods:** We conducted a 2 year observational study of adult patients who were legally authorized a medical cannabis product from a single licensed producer; we captured and analyzed authorized cannabis use patterns by cannabinoid profile (tetrahydrocannabinol [THC]-dominant; cannabidiol [CBD]-dominant; and balanced (THC:CBD) and clinical outcomes using standardized outcome measures every 3 months for 12 months at a network of medical cannabis clinics in Quebec, Canada.

**Results:** We recruited 585 patients (average age 56.5 years), of whom 61% identified as female and 85% reported pain as their primary complaint. Over 12 months, there was a significant increase in the number of products authorized (*Z*=2.59, *p*=0.01). The proportion of authorizations for a THC-dominant or CBD-dominant product increased relative to the proportion of authorizations for a balanced (THC:CBD) product (all *p*<0.01). Symptom improvement over time was observed for pain, tiredness, drowsiness, anxiety, and well-being. Patients authorized THC-dominant products exhibited less symptom improvement for anxiety and well-being relative to those authorized CBD-dominant or balanced (THC:CBD) products. Medical cannabis was well tolerated across all product profiles.

**Conclusion:** These real-world data reveal changes in medical cannabis authorization patterns and suggest that symptom improvement may vary by cannabinoid profile over 12 months of follow-up.

## Introduction

The use of medical cannabis is increasing worldwide to treat a variety of symptoms related to pain, mood, and sleep.^1^ The multiple therapeutic effects from these medical cannabis products are primarily attributed to two cannabinoids, Δ^9^-tetrahydrocannabinol (THC) and cannabidiol (CBD). THC is a partial agonist at classical cannabinoid receptors 1 (CB1) and 2 (CB2),^2^ which mediate multiple physiological processes, including pain,^3^ regulation of fear and stress,^4,5^ inflammation,^6^ and sleep–wake cycles.^7^ CBD lacks appreciable functional activity at CB1 and CB2, but has >60 molecular targets, which may contribute to its anxiolytic, anti-epileptic, analgesic, and anti-inflammatory effects.^8^ Medical use of cannabinoids includes both plant-derived cannabinoid and pharmaceutical synthetic cannabinoid products. Canadian regulations under the Cannabis Act allow patients with various indications to access plant-based medical cannabis products through federally licensed producers with an authorization form from a health care practitioner.^9^

Given the nontraditional access regimen for medical cannabis products, and the wide range of product formulations and potential therapeutic applications, real-world evidence (RWE) studies are valuable to gain insight into the authorization patterns, safety, and effectiveness of medical cannabis products. Although randomized controlled trials (RCTs) continue to be the gold standard in demonstrating treatment efficacy, results of RCTs are often difficult to generalize to a broad range of patients, providers, and health care settings,^10^ which makes RWE an important, complementary option for the study of medical cannabis. Indeed, several observational studies of medical cannabis suggest its safety and effectiveness across multiple therapeutic areas;^11–14^ however, few have examined the safety and effectiveness of medical cannabis as a function of its cannabinoid content.^15,16^ Here, we describe physician authorization patterns of medical cannabis products from one Canadian licensed cannabis producer, and we observe its safety and self-reported effectiveness as a function of product profile using patients' data from a network of medical cannabis clinics.

## Materials and Methods

### Study design and setting

This is an observational study of patients who were authorized for medical cannabis treatment and followed at Santé Cannabis, a network of four medical cannabis clinics in Quebec, Canada. Patients were either referred to the clinic by their primary care provider or self-referred. At the initial clinic appointment, physicians confirmed the patient's eligibility for medical cannabis and completed an authorization form that included the total recommended daily amount of cannabis in grams. Patients were recommended specific medical cannabis products and formats based on their medical condition and the physician's judgment, and they were provided with general patient education (i.e., dosing and titration instructions). Follow-up visits occurred at 3-, 6-, 9-, and 12-months after the initial appointment. These visits served to monitor patients' health, adjust medical cannabis authorization when needed, and collect treatment safety and effectiveness data. Patients could have been authorized up to 6 different cannabis products.

This study was approved by the McGill University Institutional Review Board. A waiver of consent was required and approved by the ethics committee and by *La commission d'accès à l'information* of Quebec.

### Population

**FIG. 1.**
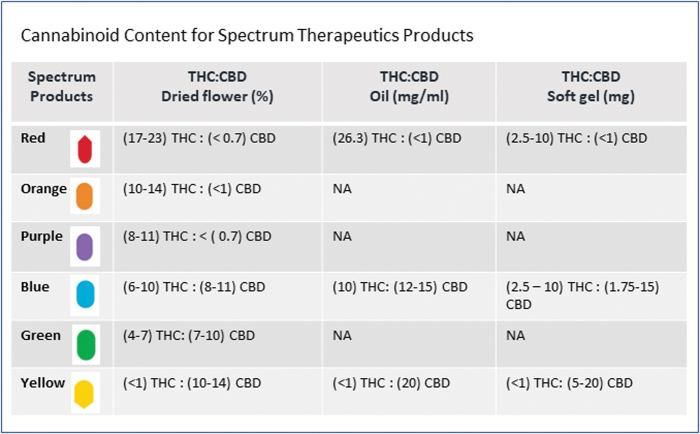
Cannabinoid content for Spectrum Therapeutics products. Color images are available online.

The sample was composed of adults (≥18 years of age) who were authorized a medical cannabis product from Spectrum Therapeutics during their initial visit between October 2017 and August 2019. Restriction of subjects to those receiving medical cannabis from a single cannabis producer was done to minimize variability of products and cannabinoid profiles between producers. Cannabis products are categorized based on cannabinoid content, reported as THC and CBD levels. Dried flower cannabis is reported in percent per gram weight of dried flower; soft gels are reported in mg per capsule; and oils mg/mL of oil. For a full list of products offered by Spectrum Therapeutics at the time of the study, please refer Figure 1.

### Data source

The following data were collected using patients' electronic medical records: demographics, medical condition(s), primary and secondary symptoms being treated, physician-authorized treatment regimen (product type, format, dose, and frequency), adverse events (AEs), patient-reported outcomes, and clinical notes.

### Outcomes

Outcomes were assessed at baseline and at the 3-, 6-, 9-, and 12-month follow-up in-person appointments. Physician authorization patterns were assessed in terms of: (1) number of products authorized, (2) format (oils and softgels vs. flowers), and (3) type defined as cannabinoid profile: THC-dominant, CBD-dominant, or balanced (relatively equal ratio of THC and CBD). To assess safety, AEs were collected and categorized by Santé Cannabis. All AEs were subsequently evaluated on completion of data collection by Canopy Growth's Pharmacovigilance department (CGC PV) and coded using the Medical Dictionary for Regulatory Activities (MedDRA) version 23 into System Organ Class (SOC) and Preferred Term (PT). To assess effectiveness, the Edmonton Symptom Assessment Scale (ESAS) measured the self-reported intensity of nine symptoms: pain, tiredness, nausea, depression, anxiety, drowsiness, appetite, well-being, and shortness of breath, on an 11-point scale (0=no symptom, 10=worst possible symptom).^17^ Although this outcome measure was developed for use in palliative care cancer patients, it is used at Santé Cannabis because its patients tend to seek relief for the wide range of symptoms assessed by this measure.

### Statistical analyses

Generalized linear mixed effects models, with fixed linear effects of time (follow-up visit), analyzed the number of authorized products, which were assumed to follow a Poisson probability distribution. Models implemented a logarithmic link function and included random intercepts and slopes.

A baseline category logit multinomial generalized estimating equation model evaluated type of product authorized at baseline and over time.^18^ The balanced (THC:CBD) products profile served as the baseline category, and analyses evaluated differences in baseline and changes over time in the relative proportion of participants using balanced (THC:CBD) products versus CBD-dominant and THC-dominant products.

There was substantial zero inflation on all ESAS measures, reflecting the mixed clinical presentation of the sample (0=no symptom). To account for this, two-part mixed effect hurdle regression models were fit.^19^ All models included random intercepts and slopes for time on the semi-continuous part and random intercepts on the zero part, with the exception of the model exploring the ESAS item of pain, for which the random intercept on the zero part was removed to achieve model convergence. Semi-continuous scores were log transformed due to positive skew. Fixed effects of time, product profile, and the interaction between time and product profile were predictors on the semi-continuous and zero part. Product profile was a time-varying covariate, with the CBD-dominant product serving as the reference category.

There was a high rate of attrition over the 12-month follow-up period. Statistical techniques were used to allow for data to be missing at random, but parameter estimates may have been biased if data were missing not at random. Two sets of sensitivity analyses to evaluate the robustness of findings to the missing data mechanism were run. First, sensitivity analyses evaluated whether missingness was predicted by observed baseline variables (age, gender, occupational status, and primary symptom) and incorporated variables that predicted missingness into all primary analysis models to strengthen the missing at random assumption. The incorporation of primary diagnosis of headache and gastrointestinal disorder as covariates did not change the significance of any primary parameter of interest. Second, sensitivity analyses restricted the analyses to only the first three time points, recognizing that sample sizes were small at the 9- and 12-month follow-ups. After limiting analyses to only three time points, there was no longer a significant decrease over time in the score on the ESAS symptom of drowsiness among CBD-dominant product users.

R version 3.6.1 was used for all analyses.^20^

## Results

### Participants

Of the 639 patients authorized Spectrum Therapeutics products between October 2017 and August 2019, 585 (92%) were ≥18 years and were included in the analyses. Of the 585 patients with data at the baseline appointment 243 (42%), 147 (25%), 88 (15%), and 42 (7%) provided data at the 3-, 6-, 9-, and 12-month follow-ups, respectively. Table 1 presents patient characteristics.

**Table 1.** Patient Characteristics, *N*=585

Baseline characteristics	*n* (%)
Female	365 (61)
Age (mean [SD])	56.5 [15.5]
Age range (years)	18–93
Occupational status	
Retired	210 (36)
On short- or long-term disability	157 (27)
Employed full- or part-time	152 (26)
Unemployed	35 (6)
Unknown	23 (4)
Student	6 (1)
Cannabis use history	
Had used dried flower	374 (64)
Had used oils	157 (27)
Primary symptom	
Pain	500 (85)
Mental health	34 (6)
Insomnia	18 (3)
Other (e.g., migraines, tremors)	16 (3)
Weight loss	6 (1)
Nausea	4
Fatigue	3
Seizures	3
Spasticity	1
Secondary symptom (any)	562 (96)

### Physician authorization patterns

At baseline, 549 (94%) patients were authorized at least one oil product, and by the 3-month follow-up, 100% of patients who remained in the study were authorized at least one oil product. There was a significant increase in the number of products authorized by physicians over time (*Z*=2.59, *p*=0.01). Physicians increased the number of authorized products by 5% at each subsequent 3-month visit, with patients authorized an estimated 1.70 products at baseline and 2.05 products at the 12-month follow-up.

**FIG. 2.**
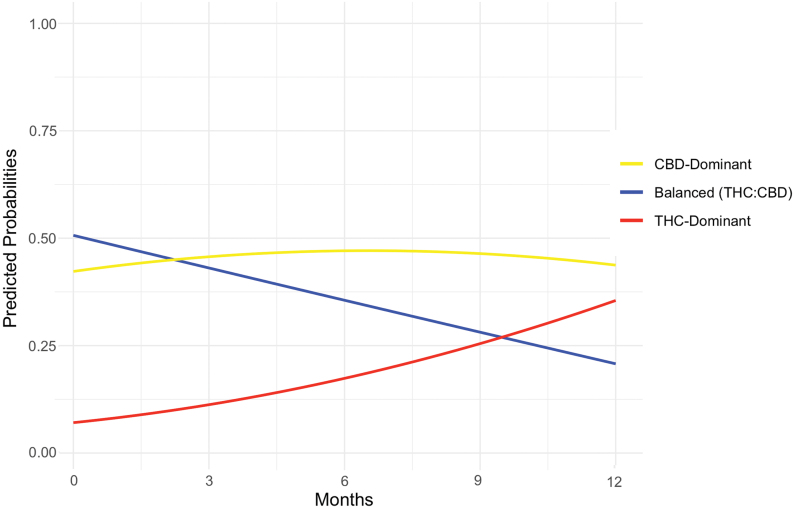
Model predicted probabilities of product profile use over time. Relative to the balanced (THC:CBD) group, there was an increase in the proportion of participants authorized a THC-dominant (*Z*=7.41, *p*<0.001, OR=1.87) or CBD-dominant (*Z*=3.26, *p*=0.001, OR=1.26) product over time. CBD, cannabidiol; OR, odds ratio; THC, tetrahydrocannabinol. Color images are available online.

Product profiles varied over time, with only 14% of patients remaining on the same product profile from baseline to the 12-month follow-up. At baseline, physicians were significantly less likely to authorize a THC-dominant (*Z*=−13.86, *p*<0.001, odds ratio [OR]=0.14) or CBD-dominant (*Z*=−2.07, *p*=0.038, OR=0.84) product than a balanced (THC:CBD) product. However, over time, the proportion of authorizations for a THC-dominant (*Z*=7.41, *p*<0.001, OR=1.87) or CBD-dominant (*Z*=3.26, *p*=0.001, OR=1.26) product increased significantly relative to the proportion of authorizations for a balanced (THC:CBD) product. This largely reflected a substantial decline in the authorization of balanced (THC:CBD) products and an increase in the authorization of THC-dominant products over time, with CBD-dominant product authorization remaining largely stable (Fig. 2).

### Safety

A total of 226 AEs were reported across 114 patients. No serious AEs were reported over the 12-month follow up period. Four AEs were assessed as serious by CGC's PV department due to a lack of confirmation on the experienced event (reported as dizziness/fainting). Table 2 shows all AEs stratified by product profile, distributed across 12 SOC categories. The proportion of AEs classified by SOC appeared similar across the three product profiles. The most frequently reported PT AEs were: somnolence (13%), dizziness (8%), nausea (6%), dry mouth (6%), and euphoric mood (5%).

**Table 2.** Adverse Events Stratified by Product Profile

		Product profile			
MedDRA SOC & PT	Total adverse events *n* (%)	Balanced (THC:CBD) product *n* (%)	CBD-dominant product *n* (%)	THC-dominant product *n* (%)	Unknown *n* (%)
Nervous system disorders	69 (31)	37 (36)	25 (28)	5 (20)	2 (33)
Somnolence	30 (13)	14 (13)	12 (13)	3 (12)	1
Dizziness	20 (8)	11 (11)	7 (8)	1	1
Headache	8 (4)	4 (4)	3 (3)	1	0
Syncope^a^	4 (2)	4 (4)	0	0	0
Coordination abnormal	3 (1)	1	2 (2)	0	0
Disturbance in attention	1	1	0	0	0
Amnesia	1	1	0	0	0
Balance disorder	1	1	0	0	0
Aphasia	1	0	1	0	0
Psychiatric disorders	53 (23)	25 (25)	19 (21)	9 (34)	0
Euphoric mood	12 (5)	4 (4)	4 (4)	4 (15)	0
Insomnia	10 (4)	2 (2)	5 (6)	3 (12)	0
Anxiety	9 (4)	6 (6)	2 (2)	1	0
Mood change	4 (2)	2 (2)	2 (2)	0	0
Confusion state	3 (1)	1	2 (2)	0	0
Irritability	3 (1)	1	2 (2)	0	0
Depressed mood	3 (1)	3 (3)	0	0	0
Distractibility	1	1	0	0	0
Bradyphrenia	1	1	0	0	0
Eating disorder and disturbance	1	1	0	0	0
Disorientation	1	1	0	0	0
Hypervigilance	1	0	1 (1)	0	0
Tension	1	0	0	1	0
Negative thoughts	1	0	1 (1)	0	0
Aggression	1	1	0	0	0
Decreased interest	1	1	0	0	0
Gastrointestinal disorders	47 (21)	20 (20)	20 (22)	5 (19)	2 (33)
Nausea	14 (6)	6 (6)	5 (6)	2 (8)	1
Dry mouth	14 (6)	6 (6)	5 (6)	2 (8)	1
Diarrhea	6 (3)	2 (2)	4 (4)	0	0
Dyspepsia	4 (2)	3 (3)	1	0	0
Abdominal discomfort	2 (1)	0	2 (2)	0	0
Reflux gastritis	2 (1)	1	1	0	0
Gastroesophageal reflux disease	1	0	1	0	0
Flatulence	1	1	0	0	0
Mouth ulceration	1	0	1	0	0
Vomiting	1	0	0	1	0
Constipation	1	1	0	0	0
General disorders and administration site conditions	18 (8)	8 (8)	8 (8)	1 (3)	1 (16)
Fatigue	8 (4)	5 (5)	3 (3)	0	0
Feeling relaxed	3 (1)	0	3 (3)	0	0
Feeling drunk	2 (1)	1	0	1	0
Asthenia	1	0	1	0	0
Pain	1	0	0	0	1
Non-cardiac chest pain	1	1	0	0	0
Hunger	1	0	1	0	0
Feeling cold	1	1	0	0	0
Respiratory, thoracic, and mediastinal disorders	16 (7)	6 (6)	6 (6)	3 (10)	1 (16)
Cough	10 (4)	2 (2)	4 (4)	3 (12)	1
Throat irritation	2 (1)	1	1	0	0
Dyspnea	2 (1)	1	1	0	0
Oropharyngeal plaque	1	1	0	0	0
Oropharyngeal pain	1	1	0	0	0
Metabolism and nutrition disorders	6 (3)	4 (4)	0	2 (7)	0
Increased appetite	5 (2)	3 (3)	0	2	0
Decreased appetite	1	1	0	0	0
Skin and subcutaneous tissue disorders	6 (3)	1 (1)	5 (5)	0	0
Hyperhidrosis	3 (1)	1	2 (2)	0	0
Skin odor abnormal	1	0	1	0	0
Pruritus	1	0	1	0	0
Erythema	1	0	1	0	0
Cardiac disorders	4 (2)	1 (1)	2 (2)	1 (3)	0
Tachycardia	3 (1)	1	2 (2)	0	0
Palpitations	1	0	0	1	0
Eye disorders	3 (1)	1 (1)	2 (2)	0	0
Visual impairment	2 (1)	0	2 (2)	0	0
Dry eye	1	1	0	0	0
Vascular disorders	2 (1)	0	2 (2)	0	0
Hot flush	1	0	1	0	0
Hypotension	1	0	1	0	0
Ear and labyrinth disorders	1	1 (1)	0	0	0
Tinnitus	1	1	0	0	0
Reproductive system and breast disorders	1	0	1 (1)	0	0
Vulvovaginal dryness	1	0	1	0	0
Total	226	104	90	26	6

^a^ Originally classified as non-serious by Santé Cannabis, assessed by CGC as serious.

CBD, cannabidiol; MedDRA, Medical Dictionary for Regulatory Activities; *n*, number of events; PTs, Preferred Terms; SOC, System Organ Class; THC, tetrahydrocannabinol.

### Self-reported effectiveness

Significant reductions in ESAS scores (i.e., improvement in symptoms) were observed over time for domains of pain (*Z*=−2.67, *p*=0.008), tiredness (*Z*=−4.29, *p*<0.001), drowsiness (*Z*=−2.81, *p*=0.005), anxiety (*Z*=−2.51, *p*=0.012), and well-being (*Z*=−2.84, *p*=0.004), but not for nausea, lack of appetite, shortness of breath, and depression among participants authorized CBD-dominant products. These significant reductions in ESAS scores were also observed for participants authorized balanced (THC:CBD) products. Participants authorized THC-dominant products observed the same degree of significant reduction over time for pain, tiredness, and drowsiness; however, they exhibited less relief over time in anxiety (*Z*=2.36, *p*=0.019) and well-being (*Z*=2.05, *p*=0.040) relative to those authorized CBD-dominant or balanced (THC:CBD) products.

**FIG. 3.**
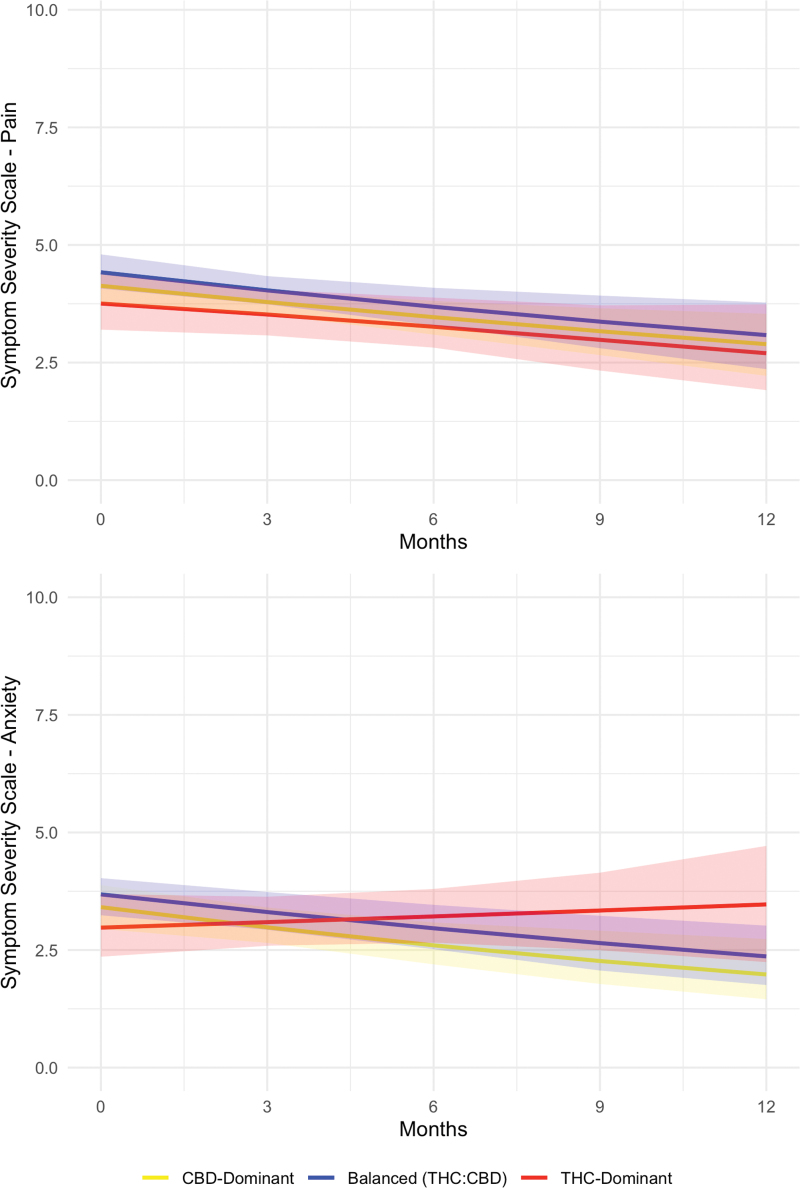
Model predicted trajectory changes in ESAS **(A)** pain, and **(B)** anxiety symptom severity scores across CBD-dominant, balanced (THC:CBD), and THC-dominant product users. Shaded regions reflect 95% confidence intervals. ESAS, Edmonton Symptom Assessment Scale. Color images are available online.

For most domains on ESAS, there was no change in the probability of patients scoring 0 (no symptom) at subsequent follow-ups. However, with regard to well-being, there was an increased probability of patients scoring 0 at subsequent follow-ups (*Z*=−2.45, *p*=0.014, OR=1.57), indicating improved well-being over time with CBD-dominant and balanced (THC:CBD). Figure 3 displays model-predicted product profile trajectories of reduction in pain and anxiety across follow-up.

## Discussion

To our knowledge, this is the first study to examine longitudinal patterns of physician authorizations of medical cannabis in adult patients. Three major findings emerged from this real-world study. First, there was significant variability in patterns of physician authorization of medical cannabis over time. Second, medical cannabis products were well tolerated. Third, medical cannabis use was associated with improvements in several psychological and physical symptoms.

Over the course of 12 months of medical cannabis treatment, oil formats consistently remained the predominant format of authorized medical cannabis, whereas other patterns of product authorization varied. Specifically, there were significant increases in the number of authorized products taken by patients over time, as well as changes in the type of product authorized. Most patients were authorized a balanced (THC:CBD) product at their initial visit, but subsequently transitioned to a THC-dominant product over time whereas CBD-dominant product authorization remained largely stable. Currently, there is an absence of concise clinical guidelines for physicians treating medical cannabis patients, and a perceived gap between current and desired knowledge levels on dosing of medical cannabis.^21^ Findings from our study suggest that, with little scientific-based evidence on how to best treat patients, physicians appear to be adopting a personalized, flexible approach to medical cannabis authorization in terms of product format and profile. Future research could examine reasons for change in product format and profile, including perceived lack of effectiveness and AEs.

Use of medical cannabis in this study was not associated with any serious AEs (as categorized by the clinics during data collection) and was generally well tolerated. These results are consistent with the results of a systematic review of 31 studies that found that 96.6% of AEs were not serious.^22^ Medical cannabis use was significantly associated with an improvement in several ESAS symptoms: wellbeing (*p*=0.004), anxiety (*p*=0.012), pain (*p*=0.008), tiredness (*p*=0.001), and drowsiness (*p*=0.005)] symptoms, and the effects of medical cannabis on two symptoms (well-being and anxiety) appeared to vary by cannabinoid content. These results align with several RWE studies that have reported improvements in overall well-being or quality of life and reduced anxiety when using medical cannabis.^11,23–26^ Moreover, clinical data from RCTs support that CBD can reduce anxiety.^27–29^

Results from the current study point to several lines of future research. Because the current study only examined product format and cannabinoid profile, a future study could investigate daily patterns of actual consumed dose (mgs of THC and CBD). Because of the complexity of medical cannabis administration (cannabinoid profile, mode of administration, product supplier), methods to capture and summarize cannabinoid dosing are desperately needed to allow for comparisons across patient groups, suppliers, and regions. Such methods could also provide more direct insight into the relationship between cannabinoid dose profile and safety and effectiveness. Future research with similar real-world designs could incorporate multiple outcome measures, including physician-reported outcome measures, to provide a more comprehensive picture of effectiveness of medical cannabis. Future research can also examine the factors that contribute to attrition from medical cannabis use over time. In one study of a cohort of medical cannabis users in Manitoba, high rates of discontinuation of prescribed cannabinoid medicines occurred within the first year of use, with duration varying by type of cannabinoid medication, age, socioeconomic status, and diagnosis.^30^ Cost may be another factor that contributes to attrition from medical cannabis treatment, as medical cannabis is currently not reimbursed by the health care system in Canada. Lack of effectiveness or AEs may also contribute to attrition. Finally, future research could examine patient adherence to medical cannabis treatment. Although treatment adherence data were not collected in the current study, in one cross-sectional study with patients who were authorized medical cannabis in Israel, adherence to medical cannabis was higher than for other medical treatments in previous studies (e.g., diabetes treatment). The patient–physician relationship and patient activation were associated with medical cannabis adherence and predicted better health outcomes and health behaviors.^31^ Data on adherence to medical cannabis treatment could inform treatment outcomes and effectiveness.

Limitations of this study were: (1) patients were recruited from only one network of medical cannabis clinics in Quebec (possible selection bias could limit generalizability of results); (2) because patients were actively under the care of a physician and providing data through the treatment clinic, data on AEs and symptom reduction could have been influenced by demand characteristics; (3) lack of a control group; and (4) participant attrition was high and reasons for loss to follow-up were unknown. In addition, there were no strategies to retain long-term patients (e.g., no participant incentives); however, sensitivity analyses suggest that the current findings are robust to the assumed missing data mechanism.

## Conclusion

In conclusion, these real-world data reveal significant changes in physician authorization practices over 12 months of treatment and show that the medical cannabis products included in the study were well tolerated. There were some variations in symptom improvement associated with use of medical cannabis by cannabinoid content and by outcome symptom. These data underscore the need to attend to product details, such as cannabinoid content, format, and dose in real-world settings providing medical cannabis treatment. Such data from RWE studies can directly inform the evaluation of causal and dose–response relationships in RCTs to better understand the effectiveness of medical cannabis.

## Abbreviations Used

AEs: adverse events
CB1: cannabinoid receptor 1
CB2: cannabinoid receptor 2
CBD: cannabidiol
CGC PV: Canopy Growth's Pharmacovigilance department
ESAS: Edmonton Symptom Assessment Scale
MedDRA: Medical Dictionary for Regulatory Activities
OR: odds ratio
PT: Preferred Term
RCTs: randomized controlled trials
RWE: real-world evidence
SOC: System Organ Class
THC: tetrahydrocannabinol
